# Detecting and monitoring arrhythmia recurrence following catheter ablation of atrial fibrillation

**DOI:** 10.3389/fphys.2015.00090

**Published:** 2015-03-27

**Authors:** Olujimi A. Ajijola, Noel G. Boyle, Kalyanam Shivkumar

**Affiliations:** UCLA Cardiac Arrhythmia Center, UCLA Health System/David Geffen School of Medicine at UCLA, University of California, Los AngelesLos Angeles, CA, USA

**Keywords:** atrial fibrillation, catheter ablation, monitoring, ambulatory, rhythm monitoring, arrhythmias, cardiac

## Abstract

Atrial fibrillation (AF) is the most common arrhythmia prompting clinical presentation, is associated with significant morbidity and mortality. The incidence and prevalence of this arrhythmia is expected to grow significantly in the coming decades. Of the available pharmacologic and non-pharmacologic treatment options, the fastest growing and most intensely studied is catheter-based ablation therapy for AF. Given the varying success rates for AF ablation, the increasingly complex factors that need to be taken into account when deciding to proceed with ablation, as well as varying definitions of procedural success, accurate detection of arrhythmia recurrence and its burden is of significance. Detecting and monitoring AF recurrence following catheter ablation is therefore an important consideration. Multiple studies have demonstrated the close relationship between the intensity of rhythm monitoring with wearable ambulatory cardiac monitors, or implantable cardiac rhythm monitors and the detection of arrhythmia recurrence. Other studies have employed algorithms dependent on intensive monitoring and arrhythmia detection in the decision tree on whether to proceed with repeat ablation or medical therapy. In this review, we discuss these considerations, types of monitoring devices, and implications for monitoring AF recurrence following catheter ablation.

## Introduction

Atrial fibrillation (AF) is the most common arrhythmia prompting presentation for clinical care (January et al., 2014). In patients over 40 years of age, the prevalence of AF is 2.3%; 6% in patients 65 years and over (Feinberg et al., 1995); and rising to 10% in those greater than 80 years (Lloyd-Jones et al., 2010) with these values expected to rise significantly in the coming decades (Go et al., 2001). Although, AF has been associated with increased mortality in the long-term (Benjamin et al., 1998), the most devastating non-fatal complication of AF remains cerebrovascular accidents (CVA) (Flegel et al., 1987; Wolf et al., 1991). To this end, the importance of anticoagulation in risk reduction of CVA, is strongly emphasized, and widely recognized (Gage et al., 2001; Wyse et al., 2002; January et al., 2014). Despite AF being one of the earliest recognized arrhythmias, treatment options for AF remain limited.

In the past two decades, few new pharmacologic options have become available, all with limited efficacy and potential toxicity. Following the recognition of pulmonary vein triggers (Haissaguerre et al., 1998), catheter ablation of AF continues to gain acceptance as a viable treatment option (Tung et al., 2012), with its main indication being the elimination of symptomatic AF, when pharmacologic agents are contraindicated or have failed (January et al., 2014).

As the decision to proceed with AF ablation depends on a number of factors including age, comorbidities, patient preference, contraindications to anticoagulation, and left atrial dimensions/fibrosis, the determination of success following ablation similarly depends on similar factors (Kircher et al., 2012), since AF is typically not considered cured following initial catheter ablation. As a result, the methods employed in detecting and monitoring AF recurrence following catheter ablation have a significant impact on the determination of “success.” Measures used to define success include complete elimination or reduction of AF burden, and the elimination or reduction of symptoms. Without adequate monitoring, the complete absence of symptoms may not equate absence of AF, and further, evaluating reduction or elimination of AF is not possible with incomplete monitoring.

### Objectives of monitoring after atrial fibrillation ablation

Success rates for catheter ablation of AF, defined typically as freedom from AF, have ranged from 60 to 90%, depending on the study, and the number of procedures performed (January et al., 2014). At present, the indication for catheter ablation of AF is symptom control in patients for whom pharmacologic options are contraindicated or have failed (Fuster et al., 2011; January et al., 2014). As such, it stands to reason that patient-reported symptoms should be the primary factor used for determination of outcome/success following ablation. This would certainly simplify follow up after catheter ablation for AF. In addition, the indication for therapeutic anticoagulation is individualized, and is based on CVA risk factors, most commonly, the CHADS2 (Gage et al., 2001) and/or CHADS2-VASc (Camm et al., 2010) scores, and is independent of the outcome following catheter ablation of AF.

However, a number of important considerations should be entertained. First, symptom reporting by patients is known to be an inaccurate estimation of true AF burden, even in patients who have experienced symptomatic episodes of AF. In a study of patients with paroxysmal or persistent AF, with implanted pacemakers for other indications, AF was detected in 88% of patients over 19 ± 11 months of follow up, with over a third of patients with long duration episodes of AF (>48 h) being asymptomatic (Israel et al., 2004). In the same study, the authors also observed poor correlation between reported symptoms and AF, with up to 40% of patients reporting symptoms consistent with AF, but no such documentation on device interrogations. In another study, up to 45% of patients with AF recurrences documented by an implanted loop recorder, reported no symptoms of AF (Tondo et al., 2013). Further, following catheter ablation for AF, the perception of symptoms may also change, either due to modulation of atrial tissue and/or innervation, or a placebo effect. In a study tracking the burden of silent AF with continuous 7-day Holter monitoring before and serially following ablation for AF, the percentage of silent episodes of AF increased significantly at 3, 6, and 12 months following catheter ablation (Hindricks et al., 2005). Intensive monitoring early (60 days) after AF ablation may be useful in identifying patients at risk of recurrence in the long-term (12-months) (Pokushalov et al., 2012), such that targeted strategies for management of such patients can be applied early, and potentially before symptoms develop. Pokushalov and colleagues employed such a strategy, and identified patients with recurrences at 3 months following AF ablation, randomizing them to medical therapy, or if possible triggers were identified with an implantable monitor, to repeat ablation (Pokushalov et al., 2011). The rate of AF recurrence at 12 months was significantly greater in patients randomized to medical therapy than those randomized to repeat ablation.

Clinical factors associated with arrhythmia recurrences may be related to the length of time post-ablation, and hence duration of follow up. In one study (Kim et al., 2014) total ablation time correlated with recurrences at 6 months. However, at 1- and 2-year follow up, other factors such as the presence of hypertension, left atrial size, left atrial appendage (LAA) emptying fraction under 20%, and decreased LAA emptying velocity were correlated with AF recurrence. This suggests that follow up monitoring and arrhythmia detection for certain patients may need to be extended past the typical 3-, 6-, and 12-month periods.

Lastly, given the variable reported success rates following catheter ablation for AF, continued research on technologies and strategies to improve AF ablation will rely heavily on accurate demonstration of no AF recurrence. It is well recognized that for patients with asymptomatic AF, the intensity of follow up is correlated with the incidence of recurrences (Roche et al., 2002). As patient symptoms may change following ablation, identification of such episodes, necessitating continued AAD use or other management strategies would be important. Accurate documentation of true AF burden following catheter ablation may also be very useful in discussions with patients. Demonstrating the impact of AF ablation or lack thereof may be useful in providing objective data to patients, and reported symptoms with AF recurrences. Such data may help patients participate in clinical decision-making regarding their arrhythmia.

### Atrial arrhythmia recurrences following catheter ablation of atrial fibrillation

Although AF recurrences following catheter ablation are most commonly due to PV reconnection (Callans et al., 2004) or incomplete ablation lines (Del Greco et al., 2008), other atrial arrhythmias may occur after AF ablation. Focal and micro-reentrant atrial tachycardias, macro-reentrant atrial flutters, and premature ventricular contractions have been reported following catheter ablation (Gerstenfeld et al., 2004; Mesas et al., 2004; Deisenhofer et al., 2006; Fiala et al., 2007; Sawhney et al., 2011; Patel et al., 2014). These arrhythmias are often complex due atrial scars created by prior ablation, and may involve multiple macro-reentrant circuits. Ablation of the critical isthmus of one circuit may shift the arrhythmia to another circuit. Of particular importance is the characterization of the recurrent arrhythmia, as this is useful in pre-procedural planning. Patients may also have more than one arrhythmia induced at electrophysiology study, and as a result, the determination of the clinical arrhythmia is crucial. Focal atrial tachycardias from pulmonary veins or other regions may be differentiated from macro-reentrant tachycardias with monitoring (Gerstenfeld et al., 2007; Chang et al., 2011; Wasmer et al., 2012), and a variety of approaches may be devised prior to electrophysiology study.

### Existing devices for rhythm monitoring

There are a variety of different cardiac rhythm monitoring devices available (Table 1) clinically for patient use today. They however fall into two major subtypes: wearable non-invasive and implanted devices.

**Table 1:** **Types of cardiac monitoring devices**.

Holter Monitor (wired and wireless devices available)
Event monitor
Presymptom memory loop recorders
Autodetect recorders
Implantable loop recorders
Pacemakers or defibrillators (already implanted)

*Source: National Institutes of Health—Public Health Information. Listed in the table are the different types of cardiac monitoring devices available for clinical use*.

#### Wearable cardiac rhythm monitors

Although multiple wearable devices exist, they can be classified by length of time for which they are worn, the number of leads, and whether real-time monitoring is performed or not. Holter monitors can be typically worn for 24 h, 7 or 30 days or for any time interval provided the device is capable of recording for that length of time. Advantages and limitations are specific to each device selected (Table 2), with the relative ease of use of short-term devices counterbalanced by low sensitivity and specificity of such devices for arrhythmia detection. Devices worn for longer periods of time, 7–30 days have the advantage of being more sensitive and specific in determining arrhythmia recurrence, however, these gains are countered by relative discomfort experienced by patients, with increasing noncompliance with wearing such devices continuously. A number of studies have assessed the diagnostic utility of different durations of monitoring (Kottkamp et al., 2004; Senatore et al., 2005; Dagres et al., 2010). Kottkamp et al. demonstrated that compared to the conventional 24-h monitoring, 7-day ECG recordings detected more arrhythmias in the early post-ablation period, and at 6 and 12 months. The yield of a longer monitoring period was underscored by the study from Senatore et al., where daily and symptom-triggered recordings transmitted trans-telephonically (for 3 months beginning 1 month post-ablation) was compared to 24-h Holter and ECG recordings. In this study, arrhythmia detection by trans-telephonic monitoring was twice that in conventional Holter and ECG recordings. Dagres and colleagues evaluated over 200 consecutive patients with 7-day Holter monitoring 6 months post-ablation. By evaluating the data collected at increasing 24-h intervals, they showed that any Holter monitoring period of less than 5 days would have significantly underestimated the rates of recurrence.

**Table 2:** **Comparison of monitoring devices for atrial fibrillation**.

	**Wearable monitors**	**Implantable monitors**
	Easily available	Prolonged monitoring period
Advantages	Inexpensive	Reliable
		Typically well-tolerated
	Limited monitoring period	Requires invasive procedure for implantation
Disadvantages	Low patient tolerance	More expensive
	Potential for noncompliance	Infection risk

*Listed are the advantages and disadvantages to the major subtypes of cardiac monitoring devices available for clinical use*.

Overcoming some of the limitations of Holter recordings, the ZIO Patch system offers improved ease of use for patients, as it is a wearable adhesive patch without interconnecting wires, and can provide continuous monitoring for 14 days. Additionally patients can shower with this system in place, but not with the standard Holter system. In a small study comparing 14-day ZIO Patch to a 24-h Holter, not surprisingly, the patch system performed better at detecting arrhythmias (Barrett et al., 2014). Other patch devices and electrodes are currently under development (Lobodzinski, 2013), with the goal of miniaturizing the devices and electrodes used for monitoring.

#### Implantable cardiac rhythm monitors

Subcutaneous implantable cardiac rhythm monitors (ICRMs) improve the sensitivity and specificity of cardiac rhythm monitoring, and overcome the limitation on patient non-compliance seen with long-term wearable devices (Table 2). If placed so that there is no interference from myopotentials, or movement of the monitor within the device pocket, implanted monitors with AF recognition algorithms are a powerful means of rhythm monitoring. In the XPECT trial, Hindricks et al. demonstrated that compared to a specialized Holter recording, ICRMs exhibited a sensitivity, specificity, positive predictive value, and negative predictive value of 96.1, 85.4, 79.3, and 97.4%, respectively, for identifying patients with any AF (Hindricks et al., 2010). However, implanted monitors are limited to only a small subset of patients who may have other indications for intensive monitoring, and have not been widely adopted for routine clinical use following AF ablation. Outside of research studies, the role that routine use of ICRMs may play in post-AF ablation rhythm management remains unclear. The recently introduced Medtronic Reveal LINQ may offer additional advantages due to its small size, reduced procedural requirements for implantation, and smaller size on the chest wall. Rather than a small incision and blunt dissection to create a pocket for the device, the LINQ is implanted by a syringe injector with an incision <1 cm. Whether the availability of such a small device and the ease of implantation will increase its use for monitoring of arrhythmias following catheter ablation outside of research studies remains to be seen. Apart from subcutaneous ICRMs, implanted permanent pacemakers (PPMs) or cardioverter-defibrillators (ICDs) with an atrial lead can also be used for monitoring recurrence following AF ablation. Due to larger memory capabilities, these devices can store more details and for longer periods than standard subcutaneous ICRMs. Another added advantage is the ability to assess the cycle lengths of AF in greater detail, as well as improved discrimination of AF from other atrial arrhythmias that may mimic AF. These devices however, have a focused applicability for patients with AF. Only patients with AF and an additional indication for pacing such as sick sinus syndrome in the case of PPMs, and cardiomyopathy or ventricular arrhythmias in the case of ICDs, will qualify for these devices.

### Our approach to monitoring recurrence following catheter ablation of atrial fibrillation

Routine monitoring following catheter ablation of AF is usually at the discretion of the physician and/or institution. At our center, patients typically undergo 7- to 14-day ambulatory telemetry monitoring during the initial evaluation and prior to catheter ablation to document AF burden. In addition to quantifying the severity of AF and correlation with symptoms, identification of other arrhythmias (narrow complex tachycardias such as atrioventricular nodal reentrant, atrioventricular reciprocating, and focal or macro-reentrant atrial tachycardias or flutters) that degenerate into AF may occur, necessitating a different ablation strategy. Following catheter ablation, patients are seen for a 30-day outpatient follow up visit during which a 12-lead ECG is performed. At 3, 6, and 12 months post-ablation, patients undergo 7-day Holter monitoring to assess AF recurrence, burden, identification of other arrhythmias, and correlation with any reported symptoms. In patients with implanted devices (pacemakers and defibrillators with an atrial lead, or loop recorders) device interrogation is typically performed to assess for atrial high rate episodes, device mode switches, and overall atrial tachycardia/AF burden.

### Implications for monitoring AF recurrences

The role of implanted monitoring devices strictly for monitoring recurrences following catheter ablation is unclear at present. Outside of research studies, routine use of ICRMs has not been reported in the literature. Routine clinical used of extended Holter monitoring for 7 days (at least 5 days) is supported by multiple clinical studies (Kottkamp et al., 2004; Piorkowski et al., 2005; Senatore et al., 2005; Dagres et al., 2010). A recent provocative study (Charitos et al., 2014) used computer simulations to predict the diagnostic utility of various durations of Holter monitoring, and the frequency with which they would need to be performed in a 12-year period. The authors reviewed the rhythm histories from 647 patients with AF and ICRMs, and used computational simulations to assess arrhythmia detection for 24-h, 7-, 14-, and 30-day monitors. The authors observed that the frequency with which any monitor needed to be performed depended on the rate and temporal aggregation of AF recurrences as well as the duration of monitoring, in the population of interest. They found that to achieve a >95% sensitivity in detecting AF recurrences, more frequent monitoring with shorter duration monitors (greater than twelve 24-h; six 7-day; four 14-day; and three 30-day monitors) were needed. When the sensitivity over the monitored time was assessed, more frequent but shorter monitoring durations were more time effective. The implication of this study is that patients are under-monitored for arrhythmia recurrence after catheter ablation, if they do not have an implanted device.

## Conclusion

The population burden of AF is expected to increase dramatically in the next decades. It will become increasingly important to adequately assess the success rates of non-pharmacologic and pharmacologic therapies to improve patient care. With the advent of smaller wearable monitoring devices, and with significant improvements in the size, implantation procedures for ICRMs, the landscape of monitoring AF recurrence following not only catheter ablation procedures, but also with pharmacologic therapies will likely significantly change.

### Conflict of interest statement

The authors declare that the research was conducted in the absence of any commercial or financial relationships that could be construed as a potential conflict of interest.
